# Plantar Pressure Detection with Fiber Bragg Gratings Sensing System

**DOI:** 10.3390/s16101766

**Published:** 2016-10-22

**Authors:** Tsair-Chun Liang, Jhe-Jhun Lin, Lan-Yuen Guo

**Affiliations:** 1 Graduate Institute of Electrical Engineering, National Kaohsiung First University of Science and Technology, Kaohsiung City 811, Taiwan; u0153801@nkfust.edu.tw; 2 Department of Sports Medicine, Kaohsiung Medical University, Kaohsiung City 807, Taiwan; yuen@kmu.edu.tw

**Keywords:** fiber-optic sensor, fiber Bragg grating, foot plantar pressure, Pearson product-moment correlation coefficient

## Abstract

In this paper, a novel fiber-optic sensing system based on fiber Bragg gratings (FBGs) to measure foot plantar pressure is proposed. This study first explores the Pedar-X insole foot pressure types of the adult-size chart and then defines six measurement areas to effectively identify four foot types: neutral foot, cavus foot, supinated foot and flat foot. The plantar pressure signals are detected by only six FBGs, which are embedded in silicone rubber. The performance of the fiber optic sensing is examined and compared with a digital pressure plate of i-Step P1000 with 1024 barometric sensors. In the experiment, there are 11 participants with different foot types to participate in the test. The Pearson correlation coefficient, which is determined from the measured results of the homemade fiber-optic plantar pressure system and i-Step P1000 plantar pressure plate, reaches up to 0.671 (*p* < 0.01). According to the measured results from the plantar pressure data, the proposed fiber optic sensing system can successfully identify the four different foot types. Measurements of this study have demonstrated the feasibility of the proposed system so that it can be an alternative for plantar pressure detection systems.

## 1. Introduction

**Figure 1:**
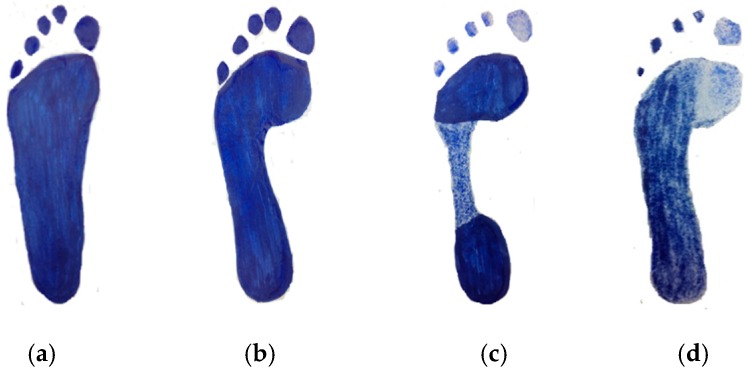
Schematic illustration of foot pressure distribution of (**a**) flat foot; (**b**) neutral foot; (**c**) cavus foot; and (**d**) supinated foot of left footprint when standing.

Not all feet are naturally identical since the human foot includes a lot of bones, joints, muscles, tendons, and ligaments. While the bones, joints, and tendons of a person differ little from that of another, a variety of different foot types come up substantially. In general, the human foot can be classified into five types: flat foot, neutral foot, pronated foot, supinated foot and cavus foot [1,2,3]. Some classification methods to identify foot type have been used in the literature [1,4,5], covering visual non-quantitative inspection, clinical values, footprint indices, and radiographic evaluation, etc. Figure 1 shows the four types of feet that are studied in this paper.

As shown in Figure 1a, the flat foot is a postural deformity, in which the arch of the foot collapses on the entire sole of the foot, having complete or near-complete contact with the ground. This foot type needs extra support in the arch region to maintain a neutral gait and to dispel the shock of running [2,6,7]. If the arch of the foot lightly collapses inward, it can be called a neutral foot. Figure 1b shows the footprint of the neutral (or normal) foot type. The neutral foot is the most common type of foot and it allows the body to naturally absorb shock [2,6,7]. If the ball and heel of the footprint are connected by a thin strip on the outside or no strip at all (see Figure 1c), this is a cavus foot. This foot type needs the most assistance with shock absorption, since the rigid structure is unable to dispel impact forces very well.

The pronated foot or eversion foot is the inward roll to distribute the impact force caused by the ground while walking or running. It is a part of the natural movements which help the lower leg deal with shock. Though this is not bad in itself, it does affect the way you run and may increase the probability of injury [2,3,6,7]. Supination or under-pronation, as depicted in Figure 1d, is the opposite of pronation and refers to the outward roll of the foot during normal motion [2].

Learning how to identify our foot type is necessary, because the foot plays a very important role in our daily life. We can choose the right shoes to support our foot type to avoid foot pain or injury when we are walking or running. Foot plantar pressure has been recognized as an important factor in the assessment of patients with diabetes and peripheral neuropathy [8,9,10,11,12]. Many papers have been published to explore the plantar pressure distribution [13,14,15,16,17,18] but have rarely addressed the application of fiber Bragg gratings (FBGs) on plantar pressure measurement [19]. This literature used three FBGs for forward and backward postural stability analysis.

In recent years, fiber-optic sensors have already been commonly utilized for numerous physical measurements [20,21,22,23,24,25,26]. The fiber-optic sensor has its intrinsic advantages of light weight, low loss, high bandwidth, and immunity to electromagnetic interference. It can be used in a variety of harsh environments and under high electric field strength to do environmental monitoring. Several fiber sensor designs based on fiber Bragg gratings (FBGs) for various applications have been reported, such as biomedical measurements [19,27], pressure [28,29,30], displacement [31,32], temperature [33,34,35], tilt [36,37] and ground vibration [38]. In this paper, we propose a fiber-optic sensor based on FBGs to detect plantar pressure. It has the advantages of a simple architecture (only using six sensing elements), low cost, temperature insensitivity, ease of classifying foot type and high stability. It can be used in the measurement of human plantar pressure distribution to classify foot type and understand whether the foot type needs to be corrected or not.

## 2. General Description and Fiber-Optic Sensor Configuration

**Figure 2:**
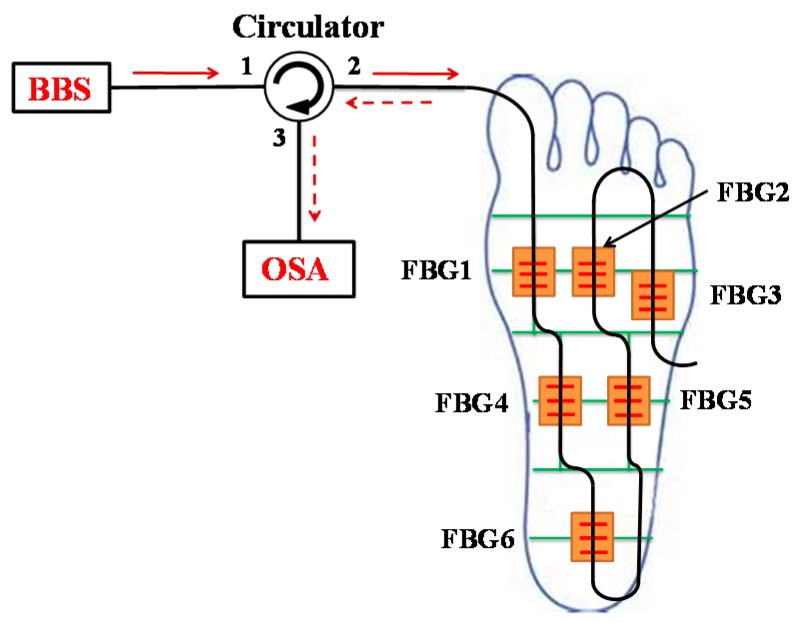
The configuration of the fiber sensing system for plantar pressure measurement.

**Figure 3:**
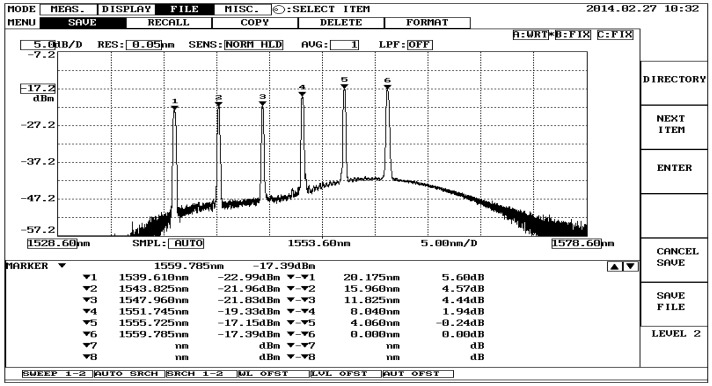
The reflection wavelength spectrum of six FBGs without any stress.

Figure 2 depicts an overview of the fiber-optic sensor measurement system used in plantar pressure detection. The output beam of a broadband source (BBS) is connected to the first port of the optical circulator (OC), and the six FBG sensors used to measure plantar pressure are connected to the second port of the OC. The BBS enters into port 1 and leaves from port 2, as shown in Figure 2. Some of the emitting light, which satisfies the Bragg condition, is reflected back to the circulator and then exits from port 3 into the optical spectrum analyzer (OSA). From the spectrum presented by the OSA, we can observe the shifts of the central wavelength of the FBGs. The sensing element of each FBG is embedded in silicone rubber, which is 3 cm long, 2 cm wide and 0.5 cm high, and placed on the insole. There are six polymer sensors in this configuration. Figure 3 shows the initial reflection wavelength spectrum of the six FBGs employed for plantar pressure measurement. The initial reflection central wavelengths are 1539.610 nm, 1543.825 nm, 1547.960 nm, 1551.745 nm, 1555.745 nm and 1559.785 nm at room temperature for FBG1, FBG2, FBG3, FBG4, FBG5, and FBG6, respectively. In addition, the sensitive length of the FBGs is equal to 1 cm. The channel spacing of the central wavelength is about 4 nm to avoid a superimposition of any two consecutive central wavelengths. When measuring the shifts of the reflection central wavelength of each FBG, we can obtain the relative plantar pressure distribution.

When a fiber grating irradiates with a broadband optical source, the grating will reflect light with a wavelength corresponding to twice the grating period (*Λ*) multiplied by the effective refractive index ($n_{\mathrm{eff}}$) of the grating in the fiber core. The central wavelength ($\lambda_{B}$) of the reflected spectral band is defined by the Bragg condition [39]:

(1) $$\lambda_{B}=2n_{\mathrm{eff}}\cdot \Lambda$$

Both the fiber refractive index ($n_{\mathrm{eff}}$) and the grating pitch (Λ) vary with the changes of strain and temperature, and consequently the Bragg wavelength is sensitive to strain and temperature. Even though the reflecting central wavelength of the sensor grating will vary with temperature slightly, the effect of temperature can be excluded. The reason is that an identification is determined by the comparative locations of the central wavelengths measured from the FBG sensors. That is, even under different temperatures, the comparative shifts of the central wavelengths are unchanged and the results identified are irrelevant to temperature.

## 3. Methods and Results

### 3.1. Subjects

**Figure 4:**
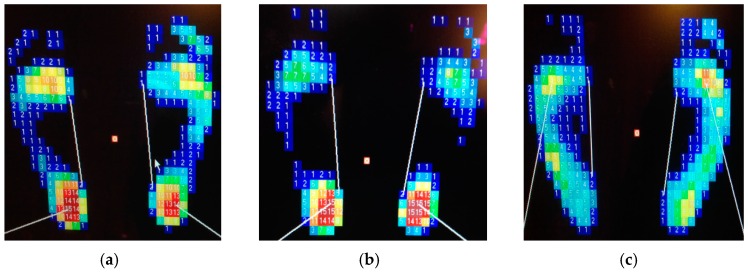

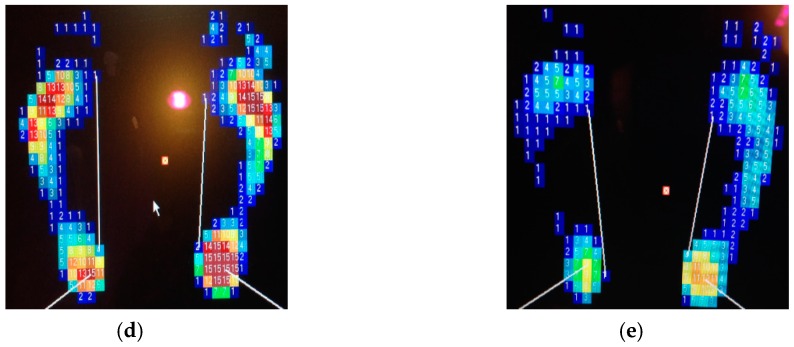
Foot plantar pressure distribution of participants: (**a**) No. 2; (**b**) No. 6; (**c**) No. 8; (**d**) No. 10; (**e**) No. 11, by i-Step P1000 digital pressure plate.

The primary aim of this work is to investigate the feasibility and functionality of the FBG sensor system to measure the plantar pressure of the foot, and thus spatially resolve the foot pressure distribution at different regions such as the forefoot, midfoot and hindfoot. Eleven subjects (nine males and two females), aged 21 to 25 years and weighing from 47 to 83 kg, were included in this work. The Institutional Review Board of Kaohsiung Medical University Chung-Ho Memorial Hospital, Taiwan, has approved this study and all subjects gave informed consent. All participants have been diagnosed to identify their foot types by the digital pressure plate of the i-Step P1000 before being subjected to FBG plantar pressure testing. The i-Step P1000 is a digital pressure plate (20 in L × 18 in W × 1.5 in H) with 1024 barometric sensors to measure the pressure exerted by the foot every 1 cm^2^. It is a current clinical foot pressure instrument and is used as a measurement reference in this study. The results are summarized in Table 1, in which there are three participants with normal feet, four with cavus feet, two with flat feet, one with supinated feet, and the other with a cavus left foot and a neutral right foot. Figure 4 shows the partial measurement results of foot pressure distribution.

### 3.2. Plantar Foot Sensing Area Definition

**Figure 5:**
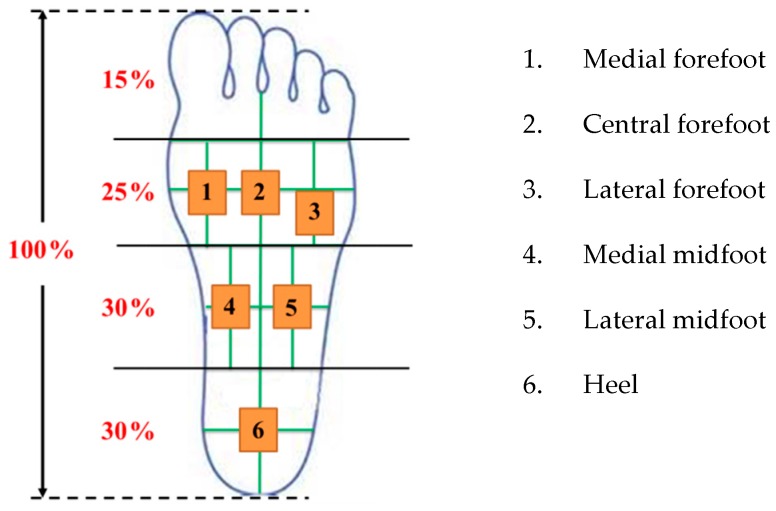
The six sensing area definitions in this research.

In this paper, plantar pressure distributions were analyzed according to the Pedar-X system in 11 healthy subjects. The foot can be subdivided into four segments of the forefoot, midfoot, hindfoot and toes. In order to set the six measurement areas, first we measured the length of the participant's foot. Then, by means of the general definition, we can get the three segments of the foot: front, middle, and hind. For the length of any one of the feet, the hindfoot length accounts for 30%, the midfoot length is 30%, the forefoot portion is 25%, and the toes account for 15% [40,41]. In accordance with the average pressure of the measurement region, we defined six sensing sub-segments [41]: hindfoot of one, midfoot of two, and forefoot of three, without the toes, as illustrated in Figure 5.

**Figure 6:**
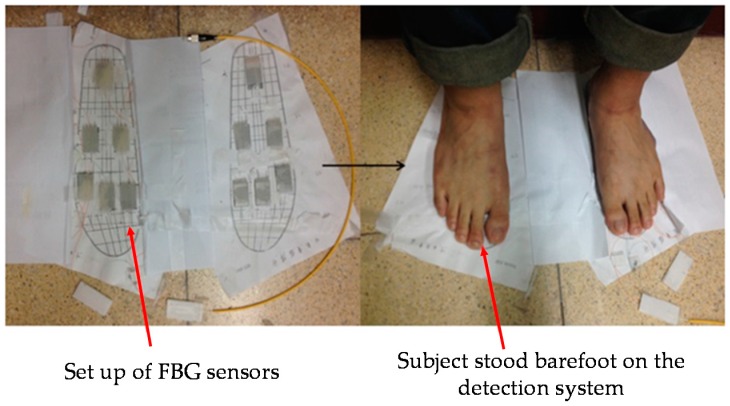
Set-up of FBG sensors and test in action.

In Figure 5, these plantar pressure sensing areas are numbered by 1 as the medial forefoot, 2 as the central forefoot, 3 as the lateral forefoot, 4 as the medial midfoot, 5 as the lateral midfoot, and 6 as the heel. When a different foot type stands on the insole, the six regions will exhibit different pressures. All subjects were asked to measure each foot five times, and then we took the average of the central wavelength shift of each FBG sensor. According to the variation of the central wavelength shift of the six FBG sensors, we can get the relative distribution of the plantar pressure and determine the subject's foot type. Figure 6 shows the prototype of the FBG sensors and a subject standing barefoot on the detection system.

### 3.3. Experimental Results

**Figure 7:**
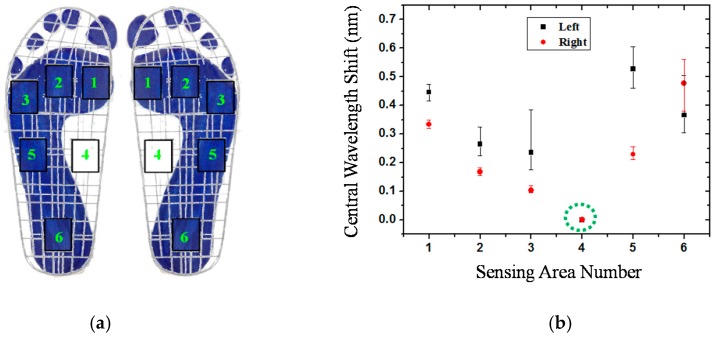
The plantar pressure distribution of the neutral feet of participant No. 2: (**a**) Footprint; (**b**) central wavelength shift as a function of sensing area number.

In order to correspond to the results of Figure 4, the measured data from the fiber-optic sensing system for the participants are shown in this section. Figure 7 shows the plantar pressure distribution of the left and right foot of the neutral feet of participant No. 2. Figure 7a depicts the footprints and Figure 7b shows the central wavelength shift of the FBGs of the six sensing areas. In the figure, the solid symbol represents the average values of five measurements of each FBG sensing element and the line represents the measurement value range of the maximum and minimum. In this work, each measured value slightly changes because the standing position and standing posture are not very consistent. From Figure 7a,b, we can obtain the plantar pressure distribution at all areas of the foot, except for the medial midfoot area. That is, there is no foot pressure that can be detected by FBG4.

**Figure 8:**
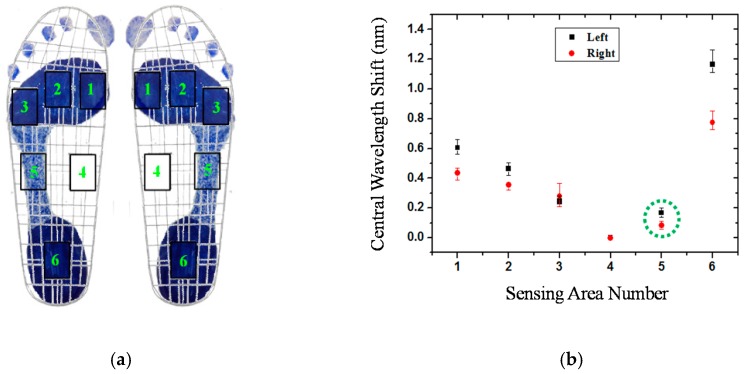
The plantar pressure distribution of the cavus feet of participant No. 6: (**a**) Footprint; (**b**) central wavelength shift as a function of sensing area number.

Figure 8 shows the plantar pressure distribution of the cavus feet of participant No. 6, one of the four participants who has cavus foot. The cavus foot is commonly known as claw foot. The cavus foot type will make the foot hollow when bearing weight. A high arch is the opposite of a flat foot. Because the arch area of the bow foot is relatively high, the pressure will be concentrated on the forefoot and heel areas. From Figure 8b, in the fifth sensing area, only a little pressure is sensed. The plantar pressure distributions are consistent among the results measured by the digital pressure plate of i-Step P1000 (Figure 4b), footprints (Figure 8a), and FBG fiber sensor (Figure 8b).

**Figure 9:**
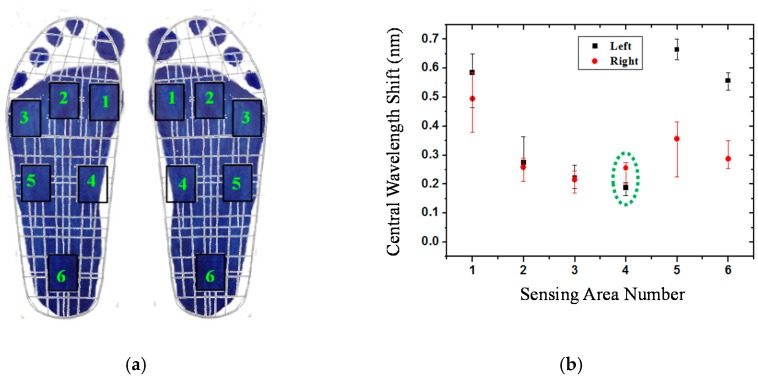
The plantar pressure distribution of the flat feet of participant No. 8: (**a**) Footprint; (**b**) central wavelength shift as a function of sensing area number.

Flat feet occur because the tissues holding the joints in the foot together (called tendons) are loose. As a result, tendons do not pull together properly; there is little or no arch. Figure 9 depicts the plantar pressure distribution of a flat foot. It differs from the others foot types; that is, there will be pressure distribution on the medial arch area, and there will be no arch shape. The measurement result of the flat foot of participant No. 8 of the fiber-optic sensing system is shown in Figure 9b. The pressure of the fourth region (the medial midfoot) with the green circle is not zero. That phenomenon is different from the other foot types, and shows a similar pressure distribution as the measured results of the digital pressure plate of i-Step P1000 (see Figure 4c).

**Figure 10:**
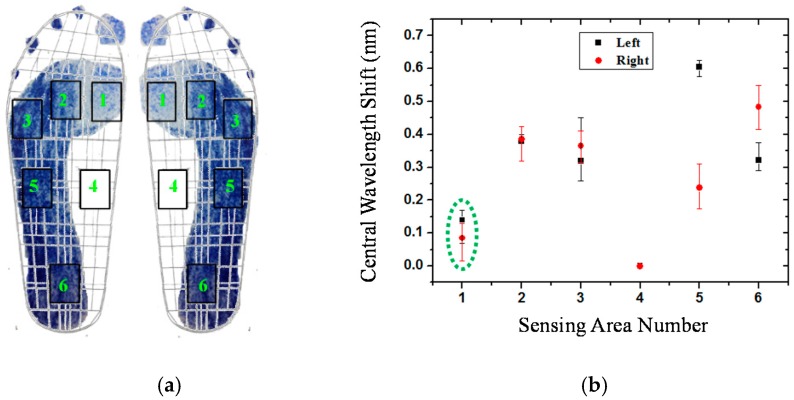
The plantar pressure distribution of supinated feet of participant No. 10: (**a**) Footprint; (**b**) central wavelength shift as a function of sensing area number.

**Figure 11:**
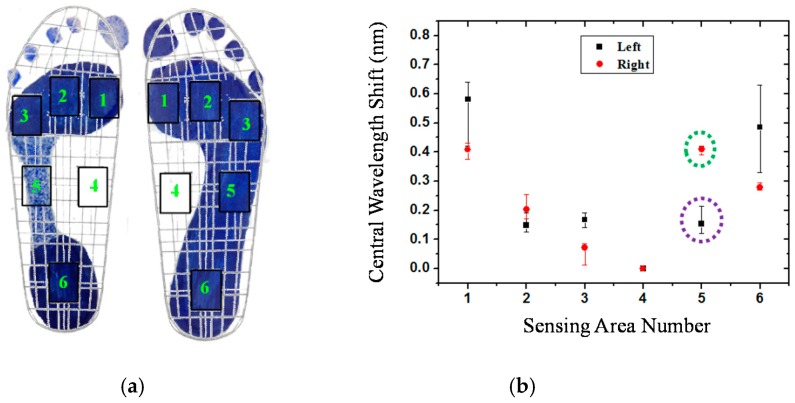
The plantar pressure distribution of participant No. 11, whose left foot is cavus and right foot is neutral: (**a**) Footprint; (**b**) central wavelength shift as a function of sensing area number.

In this work, the footprint and fiber-optic sensing result of the unique participant with supinated feet is shown in Figure 10. Supination or under-pronation is a condition that occurs when feet roll outwards, placing weight on the outside of the foot. While the foot leans to the outside, weight is distributed along the outside. A person with supinated feet will reduce the body's natural shock-absorbing capability. From Figure 10, we can see that there is no pressure detection on area 4, and area 1 only has very light pressure. This plantar pressure distribution occurs at the foot supination. Figure 11 shows the experimental results of participant No. 11. One of his feet (left foot) is pes cavus and the other is a neutral foot. From this figure, we can easily distinguish the foot types.

### 3.4. Sensing Stability

**Figure 12:**
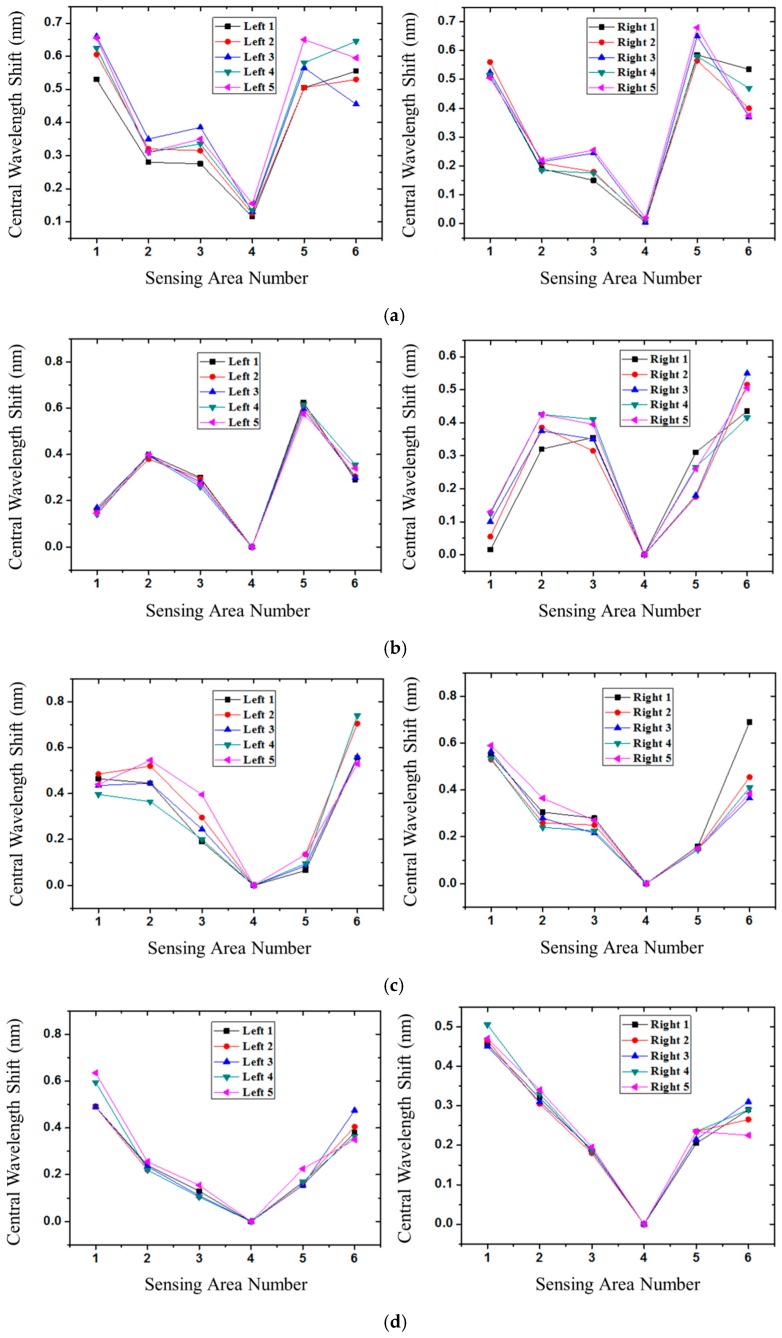
The central wavelength shift as a function of sensing area number for plantar pressure distribution of left and right foot: (**a**) Flat feet; (**b**) supinated feet; (**c**) cavus feet; and (**d**) neutral feet.

In Figure 12, we demonstrate the sensing stability of the experimental measurement results of the FBG sensors in four different foot types. Each subject’s foot was measured five times in this study. Although the result of each measurement is slightly changed due to the inconsistence of the standing position and posture each time, the overall foot pressure distribution for the foot-type classification will not be affected. If the object has flat feet, it is inevitable to detect foot pressure in the fourth sensing region (Figure 12a). The larger the foot pressure that is measured in the fourth sensing area, the more severe the flat foot situation. In contrast, there is no foot pressure in the fourth sensing area if the participant has supinated feet. At the same time, the plantar pressure is almost close to zero in the first sensing area for a supinated foot (Figure 12b). Moreover, if the participant has cavus feet, there will be little foot pressure in the fifth sensing region (Figure 12c). Of course, the sensing value of the foot pressure of the medial midfoot area is also zero. Other than the above situations, the sensitive stability of a neutral foot is shown in Figure 12d.

Some literature has discussed the abnormal plantar pressure pattern, which is related to obesity or overweight, and can be identified with its foot type by visual non-quantitative inspection. Most of the articles on plantar pressure sensors did not normalize the sensor output with respect to the single body mass value. In this work, two subjects with flat feet of different weights, 62 kg in Figure 9b and 65 kg in Figure 12a, show the non-zero pressure at the midfoot region. Two subjects with cavus feet of different weights, 71 kg in Figure 8b and 52 kg in Figure 12c, show similar pressure distribution patterns. The subject’s weight can offset the curve vertically and slightly change the curve pattern but not the foot-type classification.

### 3.5. Statistical Verification

**Figure 13:**
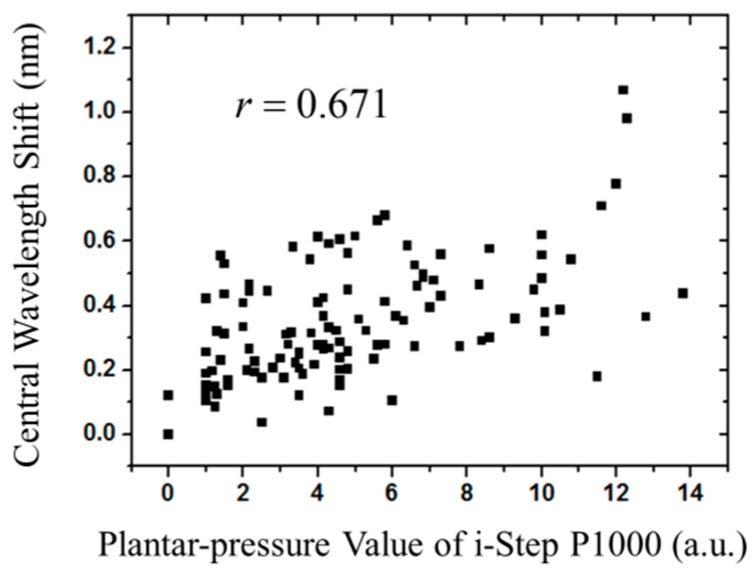
The central wavelength shift of six FBGs corresponding to the plantar pressure value of i-Step P1000.

The Pearson product-moment correlation coefficient (PPMCC or PCC) is a measure of the linear correlation between two variables [42]. A value of zero indicates that there is no association between the two variables. In order to understand the relationship of the measurement data from the digital pressure plate i-Step P1000 and the fiber-optic sensing system, we used Pearson’s correlation coefficient method. First, we calculated the average values of digital pressure on an area 3 cm × 2 cm in i-Step P1000. This area corresponds to one of the six fiber grating sensing areas, the central wavelength shift of which is measured. Figure 13 shows the correlation of all central wavelength shifts of the six FBG sensors in this study and the plantar pressure values of the digital pressure plate of i-Step P1000. Then, we calculated the Pearson correlation coefficient and got a *r*-value of 0.671 (*p* < 0.01), which reveals a positive correlation between the two variables, which can be considered sufficient for this preliminary analysis.

## 4. Conclusions

In this research, a fiber-optic sensor based on FBGs is employed to measure plantar pressure. The fiber-optic sensor system is only composed of six sensing elements. Three elements are located at the forefoot region, two at the midfoot region, and the other at the hindfoot region. The proposed system has the advantages of a simple structure, low cost, temperature insensitivity, resistance to electromagnetic interference and easy classification of foot type. A total number of 11 subjects participated in the study. All of them have been diagnosed with the digital pressure plate of i-Step P1000 before the practical examination. The experimental results validate that the performance of the proposed fiber-optic sensing system can function well for measuring plantar pressure. In addition, the six sensing areas in the proposed system have been demonstrated to be enough to distinguish various foot types. Although in the experiment there are some slight deviations among the participants, the measured results do not affect the identification of foot types.

In the proposed system, there are six sensors adopted. The flat foot type is determined by whether there is pressure detected by the fourth sensor. Then, the supinated foot can be identified under the condition that the first sensing area has the smallest value among the other five ones. If not, the fifth sensing area can verify the cavus foot, which exists if the measurement has the smallest value among the other five sensors. Otherwise, it is a neutral foot. However, the sensing areas are defined according to the normal foot. The determination rule mentioned above may be unsuitable for those with foot deformity. Most current plantar pressure sensors do not perform normalization with respect to the single body mass value. Maybe it is unnecessary because the raw plantar pressure value is more relative to clinical relevance and subjective perception. As compared with the current clinical foot pressure systems, the proposed one has the outstanding features of simple structure, low cost, immunity to electromagnetic interference and temperature insensitivity. Moreover, the proposed system can produce the reliable information of foot pressure distribution to aid foot-type classification. This work provides an alternative for plantar pressure detection systems.

## Figures and Tables

**Table 1:** The foot category of participants using the digital pressure plate i-Step 1000.

No.	Foot Type	
	Left	Right
1	neutral	neutral
2	neutral	neutral
3	neutral	neutral
4	cavus	cavus
5	cavus	cavus
6	cavus	cavus
7	cavus	cavus
8	flat	flat
9	flat	flat
10	supinated	supinated
11	cavus	neutral
